# Benzodiazepine or Antipsychotic Use and Mortality Risk Among Patients With Dementia in Hospice Care

**DOI:** 10.1001/jamanetworkopen.2025.37551

**Published:** 2025-10-14

**Authors:** Lauren B. Gerlach, Lan Zhang, Hyungjin Myra Kim, Joan Teno, Donovan T. Maust

**Affiliations:** 1 Department of Psychiatry, University of Michigan Medical School, Ann Arbor; 2 Institute for Healthcare Policy and Innovation, University of Michigan, Ann Arbor; 3 Consulting for Statistics Computing and Analytics Research, University of Michigan, Ann Arbor; 4 Center for Clinical Management Research, Veterans Affairs Ann Arbor Healthcare System, Ann Arbor, Michigan; 5 Health Services, Policy, and Practice, Brown University School of Public Health, Providence, Rhode Island

## Abstract

**Importance:**

Benzodiazepines and antipsychotics are frequently prescribed for symptom management in patients with Alzheimer disease and related dementias (ADRD) receiving hospice care, yet evidence guiding their safety and effectiveness is limited.

**Objective:**

To evaluate the association between incident benzodiazepine or antipsychotic use and 180-day mortality among nursing home residents with ADRD enrolled in hospice.

**Design, Setting, and Participants:**

This retrospective case-control study used national Medicare claims and Minimum Data Set assessments from July 1, 2014, to September 30, 2018, from US nursing homes providing long-term care. Residents with ADRD newly enrolled in hospice who had no benzodiazepine or antipsychotic use in the prior 6 months were included. Incident users were matched 1:1 to nonusers on enrollment timing, age, sex, comorbidity, cognitive function, and baseline central nervous system medication use. Follow-up was 180 days from hospice enrollment. Analyses were performed from March 2023 to December 2024.

**Exposures:**

Initiation of benzodiazepine or antipsychotic use during hospice, with no fills of the respective medication class in the prior 6 months.

**Main Outcomes and Measures:**

The primary outcome was all-cause mortality within 180 days of hospice enrollment. Hazard ratios (HRs) were estimated using Cox proportional hazards regression models adjusting for demographic, clinical, and hospice-level characteristics. Sensitivity analyses included propensity score–weighted models and models accounting for cumulative exposure.

**Results:**

Among 139 103 participants (105 372 [75.8%] female; mean [SD] age, 87.6 [7.7] years), 26 872 matched pairs were included in the benzodiazepine cohort and 10 240 in the antipsychotic cohort. For the 53 859 participants in matched cohorts, the mean (SD) age was 89.0 (6.4) years, and 45 116 (83.8%) were female. Initiation of benzodiazepine use was associated with higher 180-day mortality compared with nonuse (HR, 1.41; 95% CI, 1.38-1.44), as was initiation of antipsychotic use (HR, 1.16; 95% CI, 1.12-1.20). These findings were consistent across propensity score–weighted models and with greater cumulative exposure.

**Conclusions and Relevance:**

In this national case-control study of nursing home residents with ADRD receiving hospice care, initiation of benzodiazepine or antipsychotic use was associated with increased 180-day mortality. While these medications may provide symptom relief in appropriate clinical scenarios, their use is associated with substantial risks. These findings highlight the need for careful prescribing decisions and the development of dementia-specific hospice prescribing guidelines.

## Key Points

**Question:**

Is the initiation of benzodiazepine and antipsychotic use while enrolled in hospice associated with increased mortality among nursing home residents with Alzheimer disease and related dementias (ADRD)?

**Findings:**

In this national case-control study of 139 103 long-term nursing home residents with ADRD enrolled in hospice, new initiation of benzodiazepine or antipsychotic use while enrolled in hospice was associated with a 41% and a 16% increased risk of 180-day mortality, respectively.

**Meaning:**

These findings suggest that clinicians should carefully weigh the risks and benefits of initiating benzodiazepine and antipsychotic use for individuals with ADRD in hospice, given the potential association with increased mortality.

## Teaser

This case-control study assesses the association between incident benzodiazepine and antipsychotic prescribing and 180-day mortality among nursing home residents with Alzheimer disease and related dementias enrolled in hospice.

## Introduction

While the Medicare hospice benefit was originally designed around a cancer care model, the population it serves has changed in recent decades.^1,2^ Alzheimer disease and related dementias (ADRD) are now the most common hospice-qualifying diagnoses, rising from less than 1% of all hospice admissions in 1995 to 25% in 2023.^2,3^ However, unlike cancer, ADRD follows a prolonged and unpredictable disease trajectory, including gradual functional and cognitive decline. This makes it difficult for clinicians to determine when someone with ADRD is at the end of life and eligible for hospice enrollment. Individuals with ADRD have the longest lengths of stay of all patients with hospice-qualifying diagnoses, and 20% of those with ADRD will outlive the 6-month prognosis required for hospice eligibility and be discharged from hospice.^4,5^ Moreover, approximately 30% of hospice decedents enrolled for a condition other than ADRD also have a diagnosis of ADRD,^6^ meaning many individuals may not have advanced-stage ADRD at enrollment.

This uncertainty is particularly relevant to decisions about medication use at the end of life. Benzodiazepines and antipsychotics are commonly used in hospice to manage symptoms such as agitation, anxiety, and terminal delirium in people with ADRD, often in response to behaviors that are distressing not only to the patient but also to family caregivers and staff.^7,8,9^ While these medications can offer symptom relief, they carry risks, including falls, sedation, and confusion.^10,11,12^ The benefits of these medications may outweigh the risks for individuals in the final days or weeks of life.^13,14^ For patients earlier in the hospice trajectory—who may have months to live—the sedating effects of these drugs may compromise quality of life, impair communication, and interfere with functional independence. Furthermore, patients and families may prioritize alertness and interaction during this time.^15^ Despite these concerns, benzodiazepines and antipsychotics remain among the most commonly prescribed medications in hospice,^7,8^ and previous work has shown that individuals with ADRD enrolled in hospice are 3 times more likely to receive these medications than those not enrolled.^7^

Of the limited studies evaluating the use of benzodiazepines and antipsychotics at the end of life, none are specific to individuals with ADRD; few support antipsychotic treatment for behavioral (eg, agitated delirium)^16,17^ or physical (eg, haloperidol for treatment of nausea)^18,19^ symptoms; and some suggest these agents may even worsen outcomes.^20^ A randomized, placebo-controlled clinical trial and Cochrane review of haloperidol in terminally ill patients^20^ found that antipsychotics worsen delirium and caused earlier mortality relative to placebo. Despite the high frequency of benzodiazepine and antipsychotic use in hospice, their association with patient outcomes, particularly survival, is not well understood.^16^ One reason for this evidence gap is that hospice medication use has historically been difficult to study; the Centers for Medicare & Medicaid Services only required hospice agencies to report medications billed to the hospice benefit during a brief window from 2014 to 2018.^21^ The objective of this study was to determine the association between incident benzodiazepine and antipsychotic prescribing and 180-day mortality among nursing home residents with ADRD enrolled in hospice.

## Methods

This case-control study was approved by the Michigan Medicine Institutional Review Board, which waived the need for informed consent as data were deidentified secondary administrative claims. We followed the Strengthening the Reporting of Observational Studies in Epidemiology (STROBE) reporting guideline.

### Data Sources and Study Cohort

**Figure 1.**
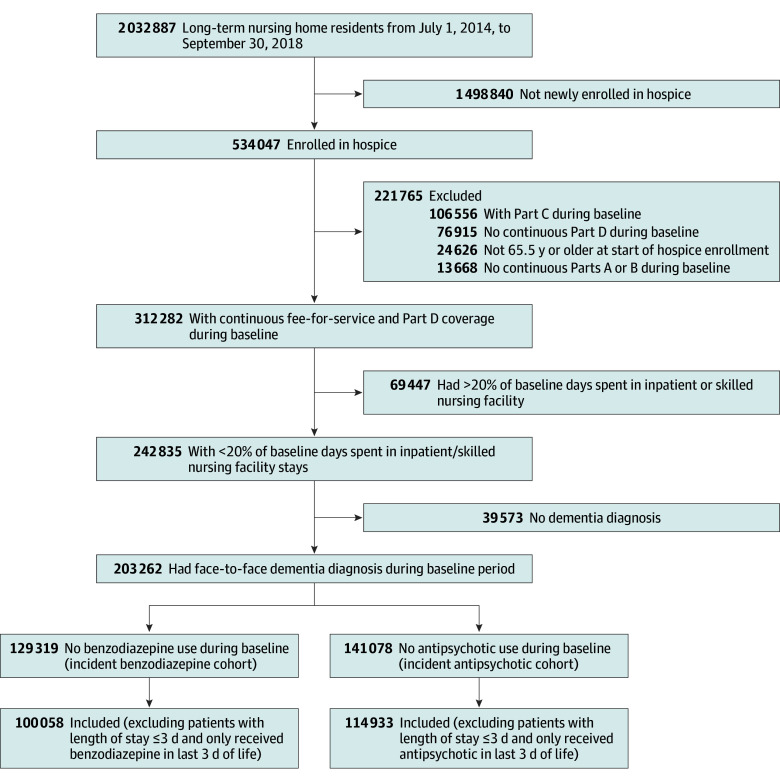
Study Flow Diagram

We conducted a retrospective new-user case-control study using a 100% Medicare cohort with dementia linked to Minimum Data Set (MDS) assessments from July 1, 2014, through September 30, 2018. This time frame was used because hospice agencies were required to report medications to Medicare from 2014 through 2018, which is the only period for which prescription records in hospice are available.^21^ The study cohort consisted of long-term nursing home residents (>100 cumulative days in a nursing home)^22^ with ADRD who were newly enrolled in hospice. We required individuals to have at least 6 months of fee-for-service Medicare enrollment prior to hospice enrollment to determine baseline characteristics, establish long-stay episodes, and ensure no prior hospice enrollment. We identified enrollees with ADRD by applying the Bynum algorithm to *International Statistical Classification of Disease, Tenth Revision*, codes on Medicare claims during the 6 months prior to hospice enrollment (sensitivity, 31.3%; specificity, 98.0%).^23,24^ We included all residents with ADRD, regardless of whether ADRD was the primary hospice-qualifying diagnosis.^1^ To capture medication exposure during the 6-month baseline, we excluded individuals with inpatient or skilled nursing facility stays of more than 20% of baseline days who would potentially have medication exposures that would not be observed in outpatient prescriptions. For benzodiazepine exposure, we further restricted the analysis to patients with no benzodiazepine use in the 6-month baseline period; similarly, for antipsychotic exposure, we restricted the analysis to those with no antipsychotic use during the baseline period. To remove residents potentially receiving the medications during the active dying period, we excluded those who received a benzodiazepine or antipsychotic only in the last 3 days of life or who were enrolled in hospice for fewer than 3 days (Figure 1).

### Exposure

Medication exposure was ascertained from the hospice revenue center file and from Part D prescription drug events for medications not covered by the hospice benefit.^25^ Decisions about medication coverage are made at the hospice medical director’s discretion, with most symptom management medications covered under the hospice benefit.^8^ The exposures of interest were incident (ie, new) use of benzodiazepines or antipsychotics during hospice enrollment. Incident use was defined as initiation of a medication during hospice enrollment with no prescription fills of that medication class in the prior 6 months. We evaluated benzodiazepine and antipsychotic use in separate parallel cohorts. The date of the first benzodiazepine prescription fill after hospice enrollment served as the index date for the benzodiazepine-exposed group; likewise, the first antipsychotic fill after enrollment was the index date for the antipsychotic-exposed group. Because days’ supply is not recorded in hospice claims, we treated any prescription fill during hospice as indicative of exposure. Patients in each cohort who did not initiate use of the medication class of interest during hospice served as the comparison group (nonusers).

### Outcomes

The primary outcome was all-cause mortality within 180 days of hospice enrollment, which was determined using hospice discharge status and dates in the Medicare hospice claims and Master Beneficiary Summary File. Observation was censored at the end of 2018, death, or discharge from hospice care, whichever occurred first.

### Demographic, Clinical, and Facility Characteristics

We captured a range of resident characteristics to control for potential confounding. Demographic characteristics included age, sex, race and ethnicity (from the Medicare Master Beneficiary Summary File and reflecting classifications assigned by the Social Security Administration, which are typically based on self-report at Social Security number application and include Hispanic, non-Hispanic Black, non-Hispanic White, and other race or ethnicity [including American Indian or Alaska Native, Asian, other, or unknown]), marital status (from the MDS assessments), and rural vs urban residence (based on zip code linkage). Race and ethnicity data were collected because prescribing patterns in hospice may vary by demographic group, reflecting systemic disparities in hospice access, symptom management, and end-of-life care preferences. Clinical characteristics included comorbidity burden (using the Gagne comorbidity score^26^), specific psychiatric diagnoses (depression, anxiety, bipolar disorder, schizophrenia, or other psychotic disorders), chronic pain, functional status (MDS Activities of Daily Living Scale^27^), and cognitive impairment (MDS Cognitive Function Scale^28^). Disruptive behaviors were identified using the MDS Distressed Behavior in Dementia Indicator,^29^ which correlates with the Cohen-Mansfield Agitation Inventory and sensitively detects clinically meaningful change in behaviors. We also identified baseline central nervous system–active medication use (any use of opioids, benzodiazepines, antipsychotics, antidepressants, antiepileptics, sedative hypnotic, or memory medications) in the 6 months prior to hospice enrollment from Part D data. Hospice agency characteristics included profit status, size, and census region.

### Statistical Analysis

To reduce confounding, we used direct 1:1 matching of incident medication users to nonusers on key covariates. We used a risk-set matching approach in which each incident user (case) was matched to a control who was at risk of starting the medication during the same time frame. Specifically, each resident who initiated use of a benzodiazepine (or antipsychotic) was matched to 1 resident who had not yet received that medication as of the initiator’s index date and who had similar hospice enrollment timing. We matched on hospice enrollment date (±7 days), age (±2 years), sex, central nervous system–active medication use (any use of opioids, benzodiazepines, or antipsychotics) during the 6-month baseline period, Gagne comorbidity score (±1 point), and Cognitive Function Scale category (exact match). Matching with replacement was used; we allowed a nonuser to serve as a match for no more than 3 cases. This created 2 matched cohorts: one for benzodiazepine initiation and one for antipsychotic initiation. Balance on additional characteristics was evaluated to confirm successful matching. After matching, we fit Cox proportional hazards regression models to estimate the association between medication initiation and 180-day mortality. The Cox proportional hazards regression models were conditioned on the matched pairs (by stratifying on matched set) and further adjusted for any residual differences in demographic, clinical, or hospice agency–level characteristics not explicitly matched. Proportional hazards assumptions were checked using Schoenfeld residuals and found to be satisfied for the exposure indicators.

We also performed several sensitivity analyses to test the robustness of our findings. First, we conducted a propensity score–matched analysis as an alternative way to control for confounding. We estimated each resident’s propensity to receive a benzodiazepine (or antipsychotic) during hospice using logistic regression including all covariates listed above. We calculated the absolute difference in propensities for all possible pairs of cases and controls, applied a caliper (<0.01), and chose the closest match for each case. To further refine the balance, we weighted Cox proportional hazards regression models by the inverse of the probability of treatment to create a population balanced on observable covariates and compared the hazard of death between medication users and nonusers.

Next, we examined outcomes among individuals whose primary hospice-qualifying diagnosis was ADRD. To assess the potential influence of concomitant new use, we performed a sensitivity analysis excluding individuals with incident new use of the other medication during hospice. Last, we examined cumulative medication exposure by incorporating the number of benzodiazepine or antipsychotic prescription fills during the first 30 days of hospice enrollment. This analysis assessed whether multiple fills (indicative of continued or repeated use) conferred different risk than a single fill. A 2-tailed *P* < .05 was used to define statistical significance. All analyses were conducted in SAS, version 9.4 (SAS Institute Inc) from March 2023 to December 2024.

## Results

### Study Cohort

A total of 139 103 participants (105 372 [75.8%] female and 33 731 [24.2%] male; mean [SD] age, 87.6 [7.7] years) were identified for the analysis. A total of 2257 participants (1.6%) were Hispanic; 13 349 (9.6%), non-Hispanic Black; 120 449 (86.6%), non-Hispanic White; and 2998 (2.2%), other race or ethnicity. The mean (SD) age of the 53 859 residents included in the matched cohorts was 89.0 (6.4) years, 45 116 (83.8%) were female, and 8743 (16.3%) were male. Most participants had severe cognitive impairment, high burden of comorbid illness, and functional dependency. We identified 100 058 nursing home residents with ADRD who were at risk of incident benzodiazepine exposure upon hospice enrollment; 47 791 (47.8%) initiated benzodiazepine use. Among 114 933 residents at risk for incident antipsychotic exposure, 15 314 (13.4%) initiated antipsychotic use during hospice (Table 1). The median time to medication initiation following hospice enrollment was 3 (IQR, 1-23) days for benzodiazepines and 3 (IQR, 1-32) days for antipsychotics. After 1:1 matching on covariates, the benzodiazepine cohort consisted of 26 872 matched pairs (26 872 initiators matched to 26 872 noninitiators), and the antipsychotic cohort consisted of 10 240 matched pairs (10 240 initiators matched to 10 240 noninitiators). Baseline characteristics were well-balanced between medication users and nonusers (eTable 1 in Supplement 1). A total of 13 219 pairs (49.2%) were enrolled in hospice for a primary hospice-qualifying diagnosis of ADRD, followed by 3685 (13.7%) for a diagnosis of stroke and 3388 (12.6%) for a diagnosis of heart disease. The mean (SD) hospice length of stay was 136.4 (186.5) days and 154.0 (197.5) days for participants with incident benzodiazepine and antipsychotic use, respectively (Table 1).

**Table 1.** Characteristics of Hospice Enrollees in Nursing Homes at Risk for Incident Benzodiazepine and Antipsychotic Prescribing^a^

Characteristic	Benzodiazepine risk (n = 100 058)		Antipsychotic risk (n = 114 933)	
	Incident prescribing (n = 47 791)	No incident prescribing (n = 52 267)	Incident prescribing (n = 15 314)	No incident prescribing (n = 99 619)
Age, mean (SD), y	87.7 (7.7)	87.7 (7.8)	87.9 (7.6)	88.2 (7.6)
Sex				
Female	35 822 (75.0)	38 826 (74.3)	11 684 (76.3)	76 627 (76.9)
Male	11 969 (25.0)	13 441 (25.7)	3630 (23.7)	22 992 (23.1)
Race and ethnicity				
Hispanic	799 (1.7)	947 (1.8)	279 (1.8)	1578 (1.6)
Non-Hispanic Black	4348 (9.1)	6383 (12.2)	1370 (8.9)	9666 (9.7)
Non-Hispanic White	41 617 (87.1)	43 555 (83.3)	13 294 (86.8)	86 220 (86.5)
Other^b^	1027 (2.1)	1382 (2.6)	371 (2.4)	2155 (2.2)
Rurality				
Urban	38 419 (80.4)	42 791 (81.9)	12 391 (80.9)	80 738 (81.0)
Rural	8050 (16.8)	8004 (15.3)	2485 (16.2)	16 184 (16.2)
Marital status				
Never married	3818 (8.0)	4894 (9.4)	1192 (7.8)	7667 (7.7)
Married	9034 (18.9)	9745 (18.6)	2757 (18.0)	18 356 (18.4)
Widowed	29 072 (60.8)	31 224 (59.7)	9517 (62.2)	62 202 (62.4)
Separated or divorced	5164 (10.8)	5682 (10.9)	1621 (10.6)	10 104 (10.1)
Other CNS medication use				
Benzodiazepine	NA	NA	4894 (32.0)	27 630 (27.7)
Antipsychotic	11 943 (25.0)	11 610 (22.2)	NA	NA
Opioid	19 050 (39.9)	19 391 (37.1)	6962 (45.5)	44 151 (44.3)
Antiepileptic	15 070 (31.5)	15 244 (29.2)	4845 (31.6)	29 775 (29.9)
Antidepressant	30 905 (64.7)	31 980 (61.2)	10 287 (67.2)	63 412 (63.7)
Sedative hypnotic	483 (1.0)	442 (0.8)	246 (1.6)	1147 (1.2)
Memory drug	20 062 (42.0)	20 757 (39.7)	6131 (40.0)	39 239 (39.4)
Gagne comorbidity scale, mean (SD)^c^	4.8 (2.6)	4.8 (2.6)	4.9 (2.6)	4.8 (2.6)
Comorbid disorders				
Depression	21 251 (44.5)	21 811 (41.7)	7208 (47.1)	42 803 (43.0)
Anxiety disorder	11 105 (23.2)	11 000 (21.0)	4801 (31.4)	27 495 (27.6)
Bipolar disorder	1689 (3.5)	1728 (3.3)	390 (2.5)	1979 (2.0)
Schizophrenia and/or psychosis	7505 (15.7)	7876 (15.1)	1727 (11.3)	9270 (9.3)
Chronic pain	36 051 (75.4)	38 958 (74.5)	11 825 (77.2)	74 853 (75.1)
MDS-ADL score^d^				
0-4	5909 (12.4)	5410 (10.4)	2128 (13.9)	10 793 (10.8)
5-8	10 134 (21.2)	9947 (19.0)	3544 (23.1)	19 758 (19.8)
9-12	12 758 (26.7)	14 066 (26.9)	4115 (26.9)	26 776 (26.9)
13-16	18 990 (39.7)	22 844 (43.7)	5527 (36.1)	42 292 (42.5)
Disruptive behaviors^e^	9057 (19.0)	8966 (17.2)	3113 (20.3)	16 423 (16.5)
MDS Cognitive Function Scale^f^				
1	3662 (7.7)	3493 (6.7)	1425 (9.3)	7640 (7.7)
2	7861 (16.4)	7857 (15.0)	2732 (17.8)	15 874 (15.9)
3	24 239 (50.7)	26 126 (50.0)	7734 (50.5)	49 146 (49.4)
4	11 544 (24.2)	14 275 (27.3)	3287 (21.5)	25 990 (26.1)
Hospice stay, mean (SD), d^g^	136.4 (186.5)	102.5 (147.5)	154.0 (197.5)	106.3 (156.7)

Abbreviations: CNS, central nervous system; MDS-ADL, Minimum Data Set Activities of Daily Living Scale; NA, not applicable.

^a^ Data are presented as No. (%) of participants unless indicated otherwise. Owing to missing data, sum totals may not equal numbers in column headings and percentages may not total 100.

^b^ Includes American Indian or Alaska Native, Asian, other, or unknown.

^c^ Scores range from −2 to 21, with higher numbers indicating more comorbidities.

^d^ Scores range from 0 to 16, with higher scores indicating greater functional dependence.

^e^ Indicates MDS Disruptive Behavior in Dementia Indicator score 1 or greater; defined by Curyto et al^29^ using MDS items for verbal and physical abuse, socially inappropriate or disruptive behavior, or resisting care.

^f^ Scores range from 1 (intact or mild impairment) to 4 (severe impairment).

^g^ For the benzodiazepine cohort, 14 077 (14.1%) had a hospice stay less than 7 days; for the antipsychotic cohort, 18 409 (16.0%) had a hospice stay less than 7 days.

### Association of Medication Initiation and 180-Day Mortality

**Figure 2.**
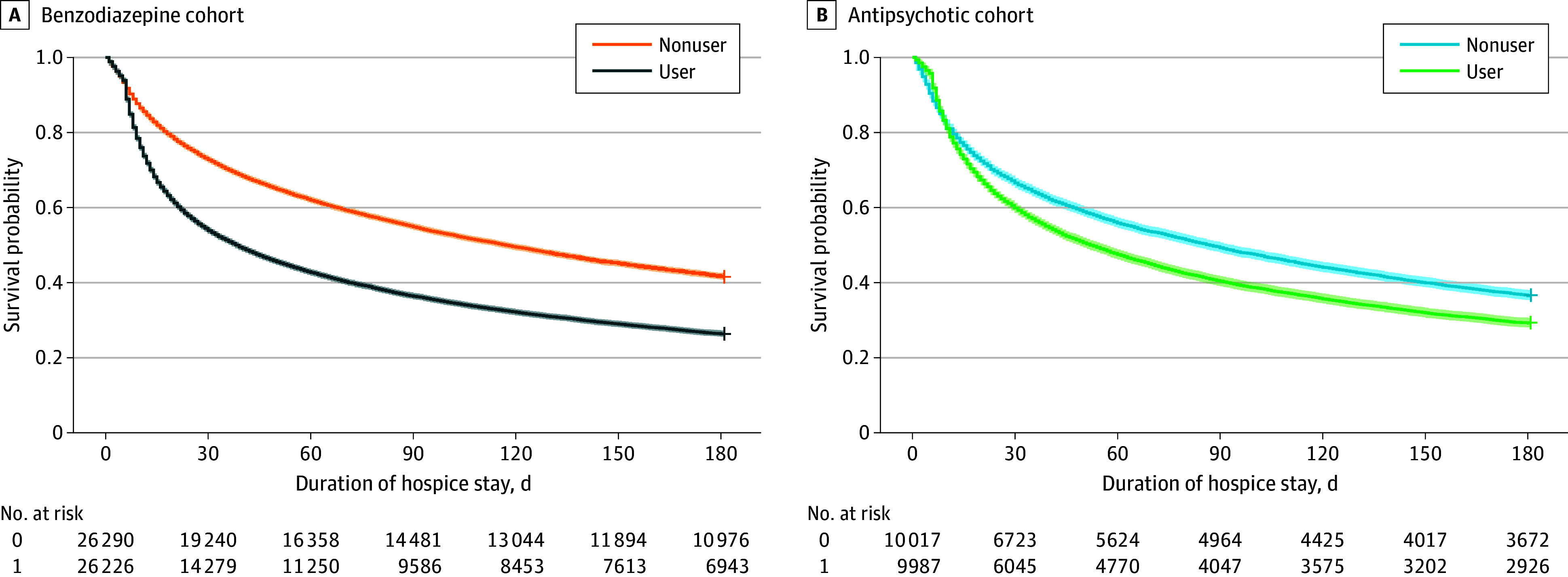
Adjusted Survival Probability by Incident Benzodiazepine or Antipsychotic Prescribing Product limit survival estimate is shown with number at risk by time.

Among participants who initiated benzodiazepine use, a greater proportion died by 180 days compared with their matched nonuser counterparts (19 283 [73.58%] vs 15 314 [58.3%], unadjusted comparison), and a similar pattern was observed for participants who initiated antipsychotic use vs nonusers (7061 [70.7%] vs 6345 [63.3]). The median number of days from the last prescription fill to death was 15 (IQR, 7-59) days for benzodiazepines and 23 (IQR, 9-92) days for antipsychotics. In Cox proportional hazards regression models accounting for direct matching and adjusting for covariates, initiation of benzodiazepine use during hospice was associated with a 41% higher hazard of death at 180 days compared with no benzodiazepine use (hazard ratio [HR] 1.41; 95% CI, 1.38-1.44) (Table 2 and Figure 2). Initiation of antipsychotic use was associated with a more modest but significant increase in mortality risk, with a 16% higher hazard of death at 180 days (HR, 1.16; 95% CI, 1.12-1.20) compared with no antipsychotic use.

**Table 2.** Risk of 180-Day Mortality for Patients With Alzheimer Disease and Related Dementias Enrolled in Hospice With Incident Benzodiazepine or Antipsychotic Use After a 6-Month Clean Period

Medication class	Direct matched cohort			Propensity score–matched cohort		
	HR (95% CI)	E value (95% CI)	*P* value	HR (95% CI)	E value (95% CI)	*P* value
Benzodiazepine	1.41 (1.38-1.44)	1.85 (1.81-1.89)	<.001	1.92 (1.88-1.96)	2.51 (2.46-2.56)	<.001
Antipsychotic	1.16 (1.12-1.20)	1.45 (1.38-1.53)	<.001	1.86 (1.79-1.94)	2.44 (2.35-2.54)	<.001

Abbreviation: HR, hazard ratio.

### Sensitivity Analyses

In propensity score–weighted Cox proportional hazards regression models, the association between initiation of medication use and mortality persisted (benzodiazepine HR, 1.92 [95% CI, 1.88-1.96]; antipsychotic HR, 1.86 [95% CI, 1.79-1.94]) (Table 2 and eTable 2 in Supplement 1). Restricting analyses to patients with a primary diagnosis of ADRD, findings were consistent with main results (benzodiazepine HR, 1.31 [95% CI, 1.27-1.36]; antipsychotic HR, 1.20 [95% CI, 1.13-1.27]). Propensity score–weighted estimates were also similar (eTable 3 in Supplement 1). When excluding individuals with incident use of the other study medication, effect estimates were similar to those of the primary analysis (benzodiazepine HR, 1.36 [95% CI, 1.33-1.39]; antipsychotic HR, 1.14 [95% CI, 1.08-1.19]) (eTable 4 in Supplement 1). Evaluating cumulative medication exposure demonstrated a similar association with mortality. For each additional prescription fill, 180-day mortality increased for both benzodiazepines (HR. 1.15; 95% CI, 1.12-1.18) and antipsychotics (HR, 1.06; 95% CI, 1.01-1.10) (eTable 5 in Supplement 1). Likewise, these results were consistent in propensity score–matched analyses as well (eTable 5 in Supplement 1).

## Discussion

In this national case-control study of nursing home residents with ADRD enrolled in hospice, initiation of benzodiazepine or antipsychotic use during hospice care was associated with a significantly increased risk of mortality within 180 days. To our knowledge, this is one of the first studies to specifically examine the survival outcomes associated with the initiation of use of these medications in hospice care for individuals with ADRD, a population for whom evidence-based prescribing guidance remains limited. The increased mortality risk associated with initiation of benzodiazepine use was substantial (41%), while that associated with initiation of antipsychotic use was more modest (16%), with findings consistent across multiple sensitivity analyses adjusting for treatment propensity and number of prescription fills. While hospice care often prioritizes comfort over longevity, these findings suggest that medication use—particularly earlier in the hospice course—may in some cases, contribute to shortened survival.

Benzodiazepines and antipsychotics are commonly used in hospice to manage agitation, anxiety, and delirium.^7,8^ However, both classes are associated with significant risks in older adults with ADRD. Antipsychotics carry a US Food and Drug Administration boxed warning for increased mortality in patients with ADRD, and benzodiazepines have been linked to respiratory suppression, sedation, falls, and aspiration.^10,11,12^ Our findings reinforce the need for careful, individualized risk-benefit evaluation prior to initiating use of these agents, particularly in patients in whom death is not imminent. While this study was not designed to estimate national prevalence, it is worth noting that nearly half of the cohort initiated use of a benzodiazepine (47.8%), and 13.4% initiated use of an antipsychotic during hospice. Initiation of antipsychotic use was lower than previously reported in other hospice settings (eg, home hospice), likely reflecting nursing home policies that monitor and penalize antipsychotic prescribing, even among hospice-enrolled residents, consistent with previous work.^7,9,30^

Our results suggest more standardized hospice prescribing guidance in ADRD may be useful. Hospice prescribing varies significantly across hospice agencies: benzodiazepine prescribing rates range from 12% to 80%, and antipsychotic prescribing rates range from 6% to 62%, even after adjusting for patient characteristics.^31,32^ Whether a patient receives a benzodiazepine or an antipsychotic in hospice may depend more on agency practice norms than clinical presentation, which is concerning, especially in light of our findings that such exposure may adversely affect survival. Hospice clinicians have conflicting views about the effectiveness and appropriateness of these medications^9,33^; in a national survey of hospice clinicians, 40% of hospice physicians believed that benzodiazepines were overused in their hospice, compared with only 8% of hospice nurses.^9,34^ Establishing national guidelines for hospice prescribing in ADRD could help to reduce unwarranted variation, promote safer practices, and ensure consistent care.

Guideline development is especially important given the challenge of prognostication in ADRD. Unlike cancer, ADRD often follows a prolonged and uncertain trajectory. Nearly 20% of hospice enrollees with ADRD outlive the 6-month prognosis required for eligibility,^4,5^ and the mean length of stay in our study exceeded 130 days. For patients who are not actively dying, patients and families may prioritize preserving cognition, communication, and function—goals that may be compromised by sedating medications. This further underscores the need for dementia-specific hospice interventions to help offer scalable, nonpharmacologic approaches, to equip hospice clinicians with effective alternatives.^35^

From 2014 to 2018, Medicare briefly required hospice agencies to report medications billed to the hospice benefit; that program has now expired, leaving hospice medication use entirely unmonitored. This represents a blind spot in oversight of end-of-life care and a gap in research and quality monitoring. Renewed efforts to track and evaluate medication use in hospice are essential to guide evidence-based practice, inform policy, and protect patient safety.

### Limitations

This study has several limitations. First, although we matched users with nonusers on observable characteristics, residual confounding from unmeasured factors is possible (eg, symptom severity). Second, our analyses relied on prescription claims, which may not fully capture actual medication use, and the hospice claims files do not contain days supplied. Third, findings are limited to Medicare fee-for-service beneficiaries in nursing homes and may not generalize to other populations (eg, patients in home hospice or those in Medicare Advantage plans). Fourth, the data reflect prescribing practices from 2014 to 2018, which may not capture more recent trends, particularly in light of national initiatives during that time aimed at reducing antipsychotic use in nursing homes. However, risks associated with medication use should not change over time. Last, this study examined all-cause mortality but did not measure symptom burden or quality of life, which are meaningful outcomes in end-of-life care.

## Conclusions

In this case-control study, initiation of benzodiazepine and antipsychotic use among nursing home residents with ADRD was associated with increased 180-day mortality. These medications are often used in hospice to manage agitation, anxiety, or physical symptoms such as nausea, but their known adverse effects—sedation, falls, aspiration, worsening cognition—combined with our finding of increased mortality risk reinforces concerns about their use in this population. This is particularly important in hospice given that decedents with ADRD in hospice are 3 times more likely to receive these medications compared with those not in hospice. Together, these data underscore the need for dementia-specific hospice prescribing guidelines, investment in nonpharmacologic alternatives, and Medicare policy reform to track and inform safe medication use in hospice.

## Notes

Supplement 1. **eTable 1.** Matching Characteristics for Benzodiazepine and Antipsychotic Cohorts **eTable 2.** Characteristics of the Propensity Score–Matching Cohort **eTable 3.** Mortality Risk Associated With Incident Benzodiazepine and Antipsychotic Use Among Hospice Enrollees With a Primary Hospice-Qualifying Diagnosis of ADRD **eTable 4.** Mortality Risk Associated With Incident Benzodiazepine and Antipsychotic Use Among Hospice Enrollees, Excluding Those With Concomitant Use of Benzodiazepines or Antipsychotics **eTable 5.** Number of Medication Fills Associated With 180-Day Mortality

Supplement 2. Data Sharing Statement
