# Supplementary material for: Benzodiazepine or Antipsychotic Use and Mortality Risk Among Patients With Dementia in Hospice Care
Source: JAMA Netw Open. 2025 Oct 14;8(10):e2537551. doi: 10.1001/jamanetworkopen.2025.37551 (PMC12522007; doi:10.1001/jamanetworkopen.2025.37551)
Supplement: Supplement 1. — eTable 1. Matching Characteristics for Benzodiazepine and Antipsychotic Cohorts eTable 2. Characteristics of the Propensity Score–Matching Cohort eTable 3. Mortality Risk Associated With Incident Benzodiazepine and Antipsychotic Use Among Hospice Enrollees With a Primary Hospice-Qualifying Diagnosis of ADRD eTable 4. Mortality Risk Associated With Incident Benzodiazepine and Antipsychotic Use Among Hospice Enrollees, Excluding Those With Concomitant Use of Benzodiazepines or Antipsychotics eTable 5. Number of Medication Fills Associated With 180-Day Mortality [file jamanetwopen-e2537551-s001.pdf]

Supplementary Online Content

Gerlach LB, Zhang L, Kim HM, Teno J, Maust DT. Benzodiazepine or antipsychotic use and mortality risk among patients with dementia in hospice care. *JAMA Netw Open.* 2025;8(10):e2537551. doi:10.1001/jamanetworkopen.2025.37551

eTable 1. Matching Characteristics for Benzodiazepine and Antipsychotic Cohorts
eTable 2. Characteristics of the Propensity Score–Matching Cohort
eTable 3. Mortality Risk Associated With Incident Benzodiazepine and Antipsychotic Use Among Hospice Enrollees With a Primary Hospice-Qualifying Diagnosis of ADRD
eTable 4. Mortality Risk Associated With Incident Benzodiazepine and Antipsychotic Use Among Hospice Enrollees, Excluding Those With Concomitant Use of Benzodiazepines or Antipsychotics
eTable 5. Number of Medication Fills Associated With 180-Day Mortality

This supplementary material has been provided by the authors to give readers additional information about their work.

© 2025 Gerlach LB et al. *JAMA Network Open*.

eTable 1. Matching Characteristics for Benzodiazepine and Antipsychotic Cohorts

	Benzodiazepine			Antipsychotic		
	**Case N= 26,872**	**Control N= 26,872**	**SD**	**Case N=10,240**	**Control N=10,240**	**SD**
**Matching variables**						
**Age, mean years**	89.24 (6.2)	89.22 (6.2)	0.002	89.05 (6.4)	89.05 (6.3)	0.001
**Sex**			0.000			0.000
Male	3,980 (14.8)	3,980 (14.8)	---	1,660 (16.2)	1,660 (16.2)	---
Female	22,892 (85.2)	22,892 (85.2)	---	8,580 (83.8)	8,580 (83.8)	---
**Baseline Medication Use**						
Benzodiazepine	---	---	---	2,641 (25.8)	2,641 (25.8)	0.00
Antipsychotic	4,296 (16.0)	4,296 (16.0)	0.000	---	---	---
Opioid	9,182 (34.2)	9,182 (34.2)	0.000	4,318 (42.2)	4,318 (42.2)	0.00
**Gagne comorbidity scale**	4.34 (2.1)	4.32 (2.0)	0.007	4.42 (2.1)	4.41 (2.0)	0.01
**Cognitive function scale**			0.000			0.00
1	731 (2.7)	731 (2.7)	---	467 (4.6)	467 (4.6)	---
2	2,906 (10.8)	2,906 (10.8)	---	1,404 (13.7)	1,404 (13.7)	---
3	16,555 (61.6)	16,555 (61.6)	---	6,097 (59.5)	6,097 (59.5)	---
4	6,680 (24.9)	6,680 (24.9)	---	2,272 (22.2)	2,272 (22.2)	---
**Variables in survival model**						
**Race/Ethnicity**			0.05			0.05
Non-Hispanic white	23,575 (87.7)	23,201 (86.3)	---	8,915 (87.1)	9,028 (88.2)	---
Non-Hispanic black	2,236 (8.3)	2,611 (9.7)	---	881 (8.6)	873 (8.5)	---
Hispanic	492 (1.8)	463 (1.7)	---	192 (1.9)	152 (1.5)	---
Other	569 (2.1)	597 (2.2)	---	252 (2.5)	187 (1.8)	---
**Rurality**			0.05			0.03
Urban	21,637 (80.5)	22,111 (82.3)	---	8,294 (81.0)	8,381 (81.9)	---
Rural	4,510 (16.8)	4,061 (15.1)	---	1,646 (16.1)	1,604 (15.7)	---
**Marital Status**			0.04			0.02
Never Married	1,767 (6.6)	2,007 (7.5)	---	686 (6.7)	710 (6.9)	---
Married	4,338 (16.1)	4,301 (16.0)	---	1,632 (15.9)	1,653 (16.1)	---
Widowed	18,047 (67.2)	17,800 (66.2)	---	6,852 (66.9)	6,864 (67.0)	---
Separated/Divorced	2,331 (8.7)	2,412 (9.0)	---	917 (9.0)	883 (8.6)	---
**Other CNS medication use**						
Antiepileptics	7,462 (27.8)	7,091 (26.4)	0.03	2,946 (28.8)	2,815 (27.5)	0.03
Antidepressants	16,905 (62.9)	16,754 (62.4)	0.01	6,707 (65.5)	6,637 (64.8)	0.01
Z drug	194 (0.7)	204 (0.8)	0.004	139 (1.4)	112 (1.1)	0.02
Memory drug	11,562 (43.0)	11,150 (41.5)	0.03	4,235 (41.4)	4,272 (41.7)	0.01

© 2025 Gerlach LB et al. *JAMA Network Open*.

	Benzodiazepine			Antipsychotic		
	**Case N= 26,872**	**Control N= 26,872**	**SD**	**Case N=10,240**	**Control N=10,240**	**SD**
**Comorbidity**						
Depression	11,305 (42.1)	10,902 (40.6)	0.03	4,576 (44.7)	4,305 (42.0)	0.05
Anxiety disorder	5,918 (22.0)	5,331 (19.8)	0.05	2,920 (28.5)	2,628 (25.7)	0.06
Bipolar disorder	650 (2.4)	655 (2.4)	0.001	222 (2.2)	169 (1.7)	0.04
Schizophrenia, psychosis	3,241 (12.1)	3,288 (12.2)	0.005	1,068 (10.4)	1,036 (10.1)	0.01
Pain	19,892 (74.0)	19,676 (73.2)	0.02	7,838 (76.5)	7,716 (75.4)	0.03
**MDS-ADL score**			0.04			0.11
0-4	2,957 (11.0)	2,665 (9.9)	---	1,262 (12.3)	1,083 (10.6)	---
5-8	5,541 (20.6)	5,425 (20.2)	---	2,344 (22.9)	2,053 (20.1)	---
9-12	7,147 (26.6)	7,269 (27.1)	---	2,770 (27.1)	2,773 (27.1)	---
13-16	11,227 (41.8)	11,513 (42.8)	---	3,864 (37.7)	4,331 (42.3)	---
**Disruptive behaviors**	4,927 (18.3)	4,719 (17.6)	0.02	2,124 (20.7)	1,735 (16.9)	0.10

Abbreviations: CNS, Central Nervous System; MDS-ADL, Minimum Data Set-Activities of Daily Living; SD, standardized difference

© 2025 Gerlach LB et al. *JAMA Network Open*.

eTable 2. Characteristics of the Propensity Score–Matching Cohort

	**Benzodiazepine**			**Antipsychotic**		
	**Case N= 29,249**	**Control N= 29,249**	**SD**	**Case N= 14,482**	**Control N=14,482**	**SD**
**Age, mean years**	87.5 (7.8)	87.5 (7.7)	0.01	87.9 (7.6)	88.0 (7.7)	0.02
**Sex**			0.09			0.13
Male	7,772 (26.6)	6,970 (23.8)	---	3,435 (23.7)	2,823 (19.5)	---
Female	21,477 (73.4)	22,279 (76.2)	---	11,047 (76.3)	11,659 (80.5)	---
**Race/Ethnicity**			0.04			0.03
Non-Hispanic white	24,747 (84.6)	25,134 (85.9)	---	12,580 (86.9)	12,682 (87.6)	---
Non-Hispanic black	3,230 (11.0)	2,949 (10.1)	---	1,297 (9.0)	1,210 (8.4)	---
Hispanic	533 (1.8)	489 (1.7)	---	260 (1.8)	284 (2.0)	---
Other	739 (2.5)	677 (2.3)	---	345 (2.4)	306 (2.1)	---
**Rurality**			0.03			0.06
Urban	23,582 (80.6)	23,895 (81.7)	---	11,696 (80.8)	12,044 (83.2)	---
Rural	4,803 (16.4)	4,528 (15.5)	---	2,364 (16.3)	2,074 (14.3)	---
**Marital Status**			0.02			0.05
Never Married	2,684 (9.2)	2,562 (8.8)	---	1,141 (7.9)	1,050 (7.3)	---
Married	5,822 (19.9)	5,746 (19.7)	---	2,665 (18.4)	2,521 (17.4)	---
Widowed	17,371 (59.4)	17,635 (60.3)	---	9,115 (62.9)	9,480 (65.5)	---
Separated/Divorced	3,372 (11.5)	3,306 (11.3)	---	1,561 (10.8)	1,431 (9.9)	---
**Other CNS medication use**						
Benzodiazepine	---	---	---	4,590 (31.7)	4,495 (31.0)	0.01
Antipsychotic	7,036 (24.1)	6,892 (23.6)	0.01	---	---	---
Opioid	11,246 (38.5)	10,685 (36.5)	0.04	6,584 (45.5)	6,253 (43.2)	0.05
Antiepileptics	9,165 (31.3)	8,776 (30.0)	0.03	4,608 (31.8)	4,429 (30.6)	0.03
Antidepressants	18,463 (63.1)	18,640 (63.7)	0.01	9,713 (67.1)	9,918 (68.5)	0.03
Z drug	279 (1.0)	253 (0.9)	0.01	229 (1.6)	265 (1.8)	0.02
Memory drug	11,944 (40.8)	12,247 (41.9)	0.02	5,786 (40.0)	5,909 (40.8)	0.02
**Gagne comorbidity scale**	4.80 (2.58)	4.56 (2.46)	0.09	4.84 (2.61)	4.50 (2.43)	0.13
Depression	12,643 (43.2)	12,489 (42.7)	0.01	6,798 (46.9)	6,840 (47.2)	0.01
Anxiety disorder	6,618 (22.6)	6,269 (21.4)	0.03	4,552 (31.4)	4,426 (30.6)	0.02
Bipolar disorder	1,004 (3.4)	928 (3.2)	0.02	368 (2.5)	317 (2.2)	0.02
Schizophrenia, psychosis	4,691 (16.0)	4,670 (16.0)	0.01	1,619 (11.2)	1,796 (12.4)	0.04
Pain	21,720 (74.3)	21,587 (73.8)	0.01	11,151 (77.0)	11,139 (76.9)	0.002
**Cognitive function scale**			0.03			0.06
1	2,106 (7.2)	2,018 (6.9)	---	1,350 (9.3)	1,128 (7.8)	---

© 2025 Gerlach LB et al. *JAMA Network Open*.

	Benzodiazepine			Antipsychotic		
	**Case N= 29,249**	**Control N= 29,249**	**SD**	**Case N= 14,482**	**Control N=14,482**	**SD**
2	4,747 (16.2)	4,509 (15.4)	---	2,578 (17.8)	2,564 (17.7)	---
3	14,636 (50.0)	14,731 (50.4)	---	7,362 (50.8)	7,425 (51.3)	---
4	7,760 (26.5)	7,991 (27.3)	---	3,192 (22.0)	3,365 (23.2)	---
**MDS-ADL score**			0.03			0.02
0-4	3,332 (11.4)	3,288 (11.2)	---	1,955 (13.5)	1,914 (13.2)	---
5-8	5,744 (19.6)	5,878 (20.1)	---	3,330 (23.0)	3,383 (23.4)	---
9-12	7,966 (27.2)	7,608 (26.0)	---	3,893 (26.9)	3,979 (27.5)	---
13-16	12,207 (41.7)	12,475 (42.7)	---	5,304 (36.6)	5,206 (36.0)	---
**Disruptive behaviors**	5,449 (18.6)	5,398 (18.5)	0.004	2,925 (20.2)	3,004 (20.7)	0.01

Abbreviations: CNS, Central Nervous System; MDS-ADL, Minimum Data Set-Activities of Daily Living; SD, standardized difference

© 2025 Gerlach LB et al. *JAMA Network Open*.

eTable 3. Mortality Risk Associated With Incident Benzodiazepine and Antipsychotic Use Among Hospice Enrollees With a Primary Hospice-Qualifying Diagnosis of ADRD

	**Direct Matched Cohort**			**Propensity Score Matched Cohort**		
**Medication Class**	**Hazard Ratio**	**95% CI**	**P-value**	**Hazard Ratio**	**95% CI**	**P-value**
**Benzodiazepine**	1.31	(1.27, 1.36)	<0.001	1.76	(1.71,1.82)	<0.001
**Antipsychotic**	1.20	(1.13, 1.27)	<0.001	1.65	(1.56, 1.75)	<0.001

N=45,281 benzodiazepine cohort, N=50,522 antipsychotic cohort

© 2025 Gerlach LB et al. *JAMA Network Open*.

eTable 4. Mortality Risk Associated With Incident Benzodiazepine and Antipsychotic Use Among Hospice Enrollees, Excluding Those With Concomitant Use of Benzodiazepines or Antipsychotics

	Direct Matched Cohort			Propensity Score Matched Cohort		
	**Hazard Ratio**	**95% CI**	**P-value**	**Hazard Ratio**	**95% CI**	**P-value**
**Benzodiazepine**	1.36	(1.33, 1.39)	<0.001	1.89	(1.84,1.93)	<0.001
**Antipsychotic**	1.14	(1.08, 1.19)	<0.001	1.62	(1.54,1.71)	<0.001

N=48,356 benzodiazepine cohort, N=9,272 antipsychotic cohort

© 2025 Gerlach LB et al. *JAMA Network Open*.

eTable 5. Number of Medication Fills Associated With 180-Day Mortality

	**Direct Matched Cohort**			**Propensity Score Matched Cohort**		
**Medication Class**	**Hazard Ratio**	**95% CI**	**P-value**	**Hazard Ratio**	**95% CI**	**P-value**
**Benzodiazepine**	1.15	(1.12, 1.18)	<0.001	1.15	(1.13,1.18)	<0.001
**Antipsychotic**	1.06	(1.01, 1.10)	0.02	1.05	(1.00, 1.11)	0.04

© 2025 Gerlach LB et al. *JAMA Network Open*.
