# Supplementary material for: Benzodiazepine or Antipsychotic Use and Mortality Risk Among Patients With Dementia in Hospice Care
Source: JAMA Netw Open. 2025 Oct 14;8(10):e2537551. doi: 10.1001/jamanetworkopen.2025.37551 (PMC12522007; doi:10.1001/jamanetworkopen.2025.37551)
Supplement: Supplement 2. — Data Sharing Statement [file jamanetwopen-e2537551-s002.pdf]

Data Sharing Statement

Gerlach. Benzodiazepine or Antipsychotic Use and Mortality Risk Among Patients With Dementia in Hospice Care. *JAMA Netw Open*. Published October 14, 2025.
doi:10.1001/jamanetworkopen.2025.37551

Data
Data available: No

Additional Information
Explanation for why data not available: The terms of the DUA with CMS do not allow these data to be shared
